# Spatial Targeting for Bovine Tuberculosis Control: Can the Locations of Infected Cattle Be Used to Find Infected Badgers?

**DOI:** 10.1371/journal.pone.0142710

**Published:** 2015-11-13

**Authors:** Catherine M. Smith, Sara H. Downs, Andy Mitchell, Andrew C. Hayward, Hannah Fry, Steven C. Le Comber

**Affiliations:** 1 UCL Department of Infectious Disease Informatics, Farr Institute of Health Informatics Research, University College London, London, United Kingdom; 2 Animal and Plant Health Agency, New Haw, Addlestone, Surrey, United Kingdom; 3 Centre for Advanced Spatial Analysis, University College London, London, United Kingdom; 4 School of Biological and Chemical Sciences, Queen Mary University of London, London, United Kingdom; The University of Tokyo, JAPAN

## Abstract

Bovine tuberculosis is a disease of historical importance to human health in the UK that remains a major animal health and economic issue. Control of the disease in cattle is complicated by the presence of a reservoir species, the Eurasian badger. In spite of uncertainty in the degree to which cattle disease results from transmission from badgers, and opposition from environmental groups, culling of badgers has been licenced in two large areas in England. Methods to limit culls to smaller areas that target badgers infected with TB whilst minimising the number of uninfected badgers culled is therefore of considerable interest. Here, we use historical data from a large-scale field trial of badger culling to assess two alternative hypothetical methods of targeting TB-infected badgers based on the distribution of cattle TB incidents: (i) a simple circular ‘ring cull’; and (ii) geographic profiling, a novel technique for spatial targeting of infectious disease control that predicts the locations of sources of infection based on the distribution of linked cases. Our results showed that both methods required coverage of very large areas to ensure a substantial proportion of infected badgers were removed, and would result in many uninfected badgers being culled. Geographic profiling, which accounts for clustering of infections in badger and cattle populations, produced a small but non-significant increase in the proportion of setts with TB-infected compared to uninfected badgers included in a cull. It also provided no overall improvement at targeting setts with infected badgers compared to the ring cull. Cattle TB incidents in this study were therefore insufficiently clustered around TB-infected badger setts to design an efficient spatially targeted cull; and this analysis provided no evidence to support a move towards spatially targeted badger culling policies for bovine TB control.

## Introduction

Bovine tuberculosis (TB) is a chronic infectious disease of cattle caused by *Mycobacterium bovis* that can also infect humans and a range of other species. Although the risk posed by *M*. *bovis* to human health in the UK is now considered negligible [1], it remains a major animal health and economic issue. The incidence of cattle TB in England has increased over the last 25 years. In 2014, there were 4,713 new herd TB incidents, compared to 1,075 in 1996, and the average cost of such an incident in a high incidence area is estimated at around £34,000 [2].

Control of bovine TB in the UK is primarily focused on surveillance of cattle herds through regular testing. Skin test positive animals are removed for slaughter, and herds placed under movement restrictions with enhanced testing until all remaining cattle have had negative tests [3]. However, the issue is complicated by the presence of a reservoir species, the Eurasian badger, *Meles meles*. In 1971, a dead badger found in Gloucestershire, an area now with high cattle TB incidence, tested positive for bovine TB. It is therefore postulated that transmission within and between badger and cattle populations is maintaining the disease in the environment [4,5]. On this assumption, various policies to control cattle TB that include culling of badgers have since been implemented [1,6–8]. Such policies are controversial because they do not discriminate between infected and uninfected badgers, and may lead to an increase in cattle TB in some circumstances [9]. The current strategy for achieving officially bovine TB free status for England includes badger culling in large licensed locations in high incidence areas [10] (Table 1).

**Table 1:** Badger culling strategies used in the current bovine tuberculosis policy in England, the Randomised Badger Culling Trial, and hypothetical spatially targeted culling strategies used in this study.

Badger culling strategy	When used	Description
**Pilot culls in Somerset and Gloucestershire**	Current policy in England	Culling by industry in licenced areas. The terms of the licences require that culling be widespread and conducted over areas at least 150 km^2^; that at least 70% of the land should be accessible for culling; that an effective cull be carried out for a minimum of four years, and that the estimated badger population must be reduced by at least 70% in the first year of the cull [10].
**Proactive culling**	Randomised Badger Culling Trial	Annual repeated culling across all accessible land (approximately 100 km^2^) in each of ten trial areas [9].
**Reactive culling**	Randomised Badger Culling Trial	Local culling on or near farmland where recent cattle TB incidents had occurred within ten trial areas [9]. Average reactive culling procedure covered 5.3 km^2^.
**Ring cull**	Hypothetical design	Culling across land in circular areas of varying radii around cattle TB incidents.
**Geographic profiling**	Hypothetical design	Culling across land in areas identified by novel geographic profiling method as likely sources of bovine TB incidents.

Targeting of badger culls to smaller geographic areas would be attractive if it reduced the economic costs of these operations and efficiently prioritised culling of infected over uninfected badgers. Close spatial correlation between badger and cattle TB implies that this is possible [11]. However, experimental evidence from the Randomised Badger Culling Trial (RBCT) has shown that localised ‘reactive’ culling can be associated with increases in cattle TB incidence [9,12–14]. Reactive culling involved removal of badgers in small areas around confirmed cattle TB incidents. Other approaches based on a ‘ring cull’ concept have also been conducted under previous policies, including the ‘clean ring’ strategy in the early 1980s [1]. More sophisticated methods of spatial targeting have not been tested.

Geographic profiling (GP) presents a new potential approach to the spatial targeting of badger culls for bovine TB control. GP is a statistical technique used to identify source locations using linked case locations, i.e. those arising from a common source. It was pioneered in criminology as a means of prioritising long lists of suspects in instances of serial crime [15], and has recently been successfully applied to analogous problems in biology. For example, it has been used to locate source populations from which invasive species have dispersed using their observed locations; and to identify the sources of infectious diseases in humans from case resident locations [16]. Applying GP to the problem of bovine TB raises the possibility of using cattle herd TB incident locations to predict the locations of setts housing TB-infected badgers.

In this study, we used historical data from the RBCT to assess a ring cull design and geographic profiling as hypothetical methods of targeting badgers infected with TB (Table 1). We aimed to: (i) Determine the effectiveness of locating setts housing TB-infected badgers using culls based on these designs and (ii) Compare the efficiency of the two methods.

## Methods

### Study sites and badger and cattle TB data

This analysis was based on data from the RBCT, a large scale field trial which ran from 1998 to 2005 and aimed to quantify the contribution of two badger culling strategies on the incidence of TB in nearby cattle [9]. The RBCT was conducted in 30 areas of England, each located in a high-risk area for cattle TB and measuring approximately 100 km^2^. The 30 areas were grouped into 10 sets of three, each called a triplet. Within each triplet, one area was subjected to approximately annual culling across all accessible land (‘proactive culling’), and in one area badgers were culled locally on or near farmland where recent outbreaks of TB had occurred in cattle (‘reactive culling’) (Table 1). The remaining area received no culling (‘survey only’) and acted as an experimental control with which the culling treatments could be compared. Treatments were assigned to trial areas at random.

Here, we have used data from the first cull in the proactive trial areas, in which badger TB status is known across the entire study region. The initial cull only was used as it was free from the potentially disruptive effects of previous widespread repeated culls conducted during the RBCT on the spatial distributions of TB in cattle and badgers. Possible effects of previous culling operations, including the Government Interim Strategy (1986–1998), were ignored because culling was conducted over small areas and generally involved removal of relatively small proportions of badgers [1].

In 2001 the operations of the RBCT were suspended due to a national foot-and-mouth disease (FMD) epidemic, causing a delay in the enrolment of three trial areas into the study. During the epidemic period, removal of infected cattle from the environment was postponed, and restrictions were placed on cattle movement. This led to increased potential for TB disease to spread between cattle and from cattle to badgers, and an increased prevalence of *M*. *bovis* in badgers was observed [17]. In this analysis we therefore used only on the seven trial areas in which the initial proactive cull took place before the FMD epidemic.

Locations of farms with cattle herds that had bovine TB breakdowns in the year prior to the initial cull were extracted from the routine herd surveillance database used at the time of the RBCT, VetNet. A breakdown is the term used to describe placement of a herd under movement restrictions following positive tuberculin skin tests from one or more animals in the herd, or the identification of infection in an animal during post-mortem inspection. The tuberculin skin test measures the relative skin reactions to injections of *M*. *bovis* and another Mycobacterium, *M*. *avium*: Animals whose reaction to the test meets defined criteria are classified as ‘reactors’ and are compulsorily slaughtered. Breakdowns are confirmed if lesions characteristic of TB are identified at post mortem or *M*. *bovis* is cultured from tissue samples. All herds within the RBCT areas were subject to annual testing and only confirmed breakdowns were included in this analysis.

Badger sett data were extracted from the RBCT database. During the trial, badger carcasses were examined and cultured for TB using a standard protocol [18]. For the purposes of this analysis, we classified badger setts as ‘infected’ or ‘uninfected’ according to the test results of badgers trapped during the initial cull. If at least one badger captured at the sett was found to be infected with TB, the sett was classified as infected; if no captured badgers were infected with TB, the sett was classified as uninfected. Setts at which no badgers were captured were excluded.

Analysis of each trial area was restricted to a region defined by the rectangular minimum bounding box enclosing the locations of the breakdowns, with a buffer zone extending the lengths of the sides by 5%.

### Methods of spatial targeting

Two methods of spatial targeting based on breakdown locations, a ring cull and GP, were used to design alternative search strategies for badger setts in each of the trial areas. Search strategies order the space within the trial areas according to the likelihood that the points are locations of infected setts. Success of the strategies at targeting setts is quantified using the hit score, the proportion of the ordered area that would have to be searched before the sett is reached. For example, a sett with a hit score of 10% would be located after searching one tenth of the study area, whilst the other 90% could remain unsearched. The smaller the hit score, the higher the probability that the location is a source.

Hit scores for setts using the ring cull approach were calculated using a ring of radius of the minimum distance from any breakdown required to include the sett. For example, a sett 1 km from the closest breakdown would require a ring of radius 1 km to be drawn around all of the breakdowns in the area to be included in the cull. The hit score of this sett is the percentage of the total area covered by all of these rings, after clipping to the shape of the trial region. To avoid counting the same area twice, rings from different breakdowns that cover overlapping regions are merged.

Here, we used the Dirichlet Process Mixture model implementation of GP, described in detail elsewhere [19]. It calculates hit scores according to an ordered probability surface defined, in short, as follows. First, a clustering algorithm is applied to the locations of breakdowns to divide them into groups that may have arisen from the same source, with breakdowns that are close together being more likely to be assigned to the same cluster. No prior assumptions are made about the number of potential sources, and therefore number of clusters produced. Following the grouping step, migration profiles, in the form of bivariate normal distributions, are defined for all possible source locations. These migration profiles describe the probability of a breakdown arising at each location, given the location of the potential source. The problem is then inverted using Bayes’ rule to calculate the probability of a source occurring in each location given the observed set of breakdowns. Finally, the solution is obtained using Markov Chain Monte Carlo methods, allowing the technique to be applied to large data sets.

An appropriate value for the prior expectation on the clustering parameter, denoted σ, must be selected to run the GP analysis. This value, measured in decimal degrees, is the standard deviation of the bivariate normal distribution of points around the source; thus, approximately 68% of cases will be expected to occur within this distance of the source, 95% within twice this distance and 99.7% within three times this distance. Selection of an appropriate value of σ is based on knowledge of the system under consideration, and, where possible, previous GP analyses.

Here, the value of σ relates to the distance over which badgers are likely to interact with, and potentially transmit TB between, cattle herds. Although there have been no previous GP analyses of this disease system to guide selection of this parameter, various studies have estimated badger ranging distances and bovine TB cluster sizes. An analysis of spatial clustering of badger and cattle TB from the RBCT found that infection in the two species was spatially associated at a scale of 1–2 km [11]. Other published data on badger home range sizes suggest that in areas with high badger density, such as the South West and West of England, badger home range size is generally small, with estimates in the region of 0.2 to 0.8 km^2^ [20,21]. Bait return studies in the RBCT survey only areas found median return distances in the range of 220–370m, consistent with these relatively small home range size estimates [9,12]. However, badger dispersal has also been demonstrated over longer distances, particularly in TB-infected badgers [22].

Given this range in badger dispersal estimates, a σ value of 0.02 decimal degrees was used for the primary analysis and sensitivity analyses were also conducted with a range of alternative values of σ. At the latitudes of the trial locations, σ of 0.02 assumes that 68% of breakdowns would occur within approximately 1700m of infected setts if all breakdowns resulted from badger to cattle transmission. This value was selected to be conservatively large, accounting for dispersal at the upper limit of likely distances.

### Statistical methods

Hit scores were converted into search areas to allow comparison between trial areas of different sizes (search area = hit score * total trial area).

We used an adaptation of survival analysis to compare the effectiveness of searches over all potential search areas. Survival analysis is a technique typically used to analyse ‘time to event’ data, for example to compare the time to death of patients in a clinical trial of a new cancer therapy versus control. The same logic can be applied to analyse other nonnegative random variables; for example, we can consider the number of incidences (infected or uninfected setts in this study, or deaths in traditional survival analysis) as a function of increases in search area (as in this study) as opposed to time (traditional survival analysis) [23]. For each additional sett included in a search area of increasing size, the spatial survival function is the proportion of setts with a minimum search area of an equal or smaller size. Spatial survival functions were calculated for each trial region separately and for data aggregated across all trials.

Graphical comparisons of survival functions were made using Kaplan-Meier (KM) curves, which show the number and proportion of setts that would be included in a search of increasing size. Cox regression analysis was used to calculate the relative rate (hazard ratio) of including setts in search areas of increasing sizes, both unadjusted and using a multilevel model that adjusted for the random effects of trial area to account for the clustered design of the study. Where necessary, the search area was split into sections over which the survivor functions were proportional and stratum-specific Cox regression estimates calculated.

Effectiveness of spatial targeting of setts with TB-infected badgers was determined by comparing sett survival functions for infected and uninfected setts; efficiency of the ring cull and GP methods were compared using the survival functions for infected setts only.

Analyses were conducted in R [24], using the packages Rgeos [25], for spatial analyses, Rgeoprofile [26], for geographic profiles, and Survival [27] and coxme [28] for survival analysis.

## Results

**Fig 1:**
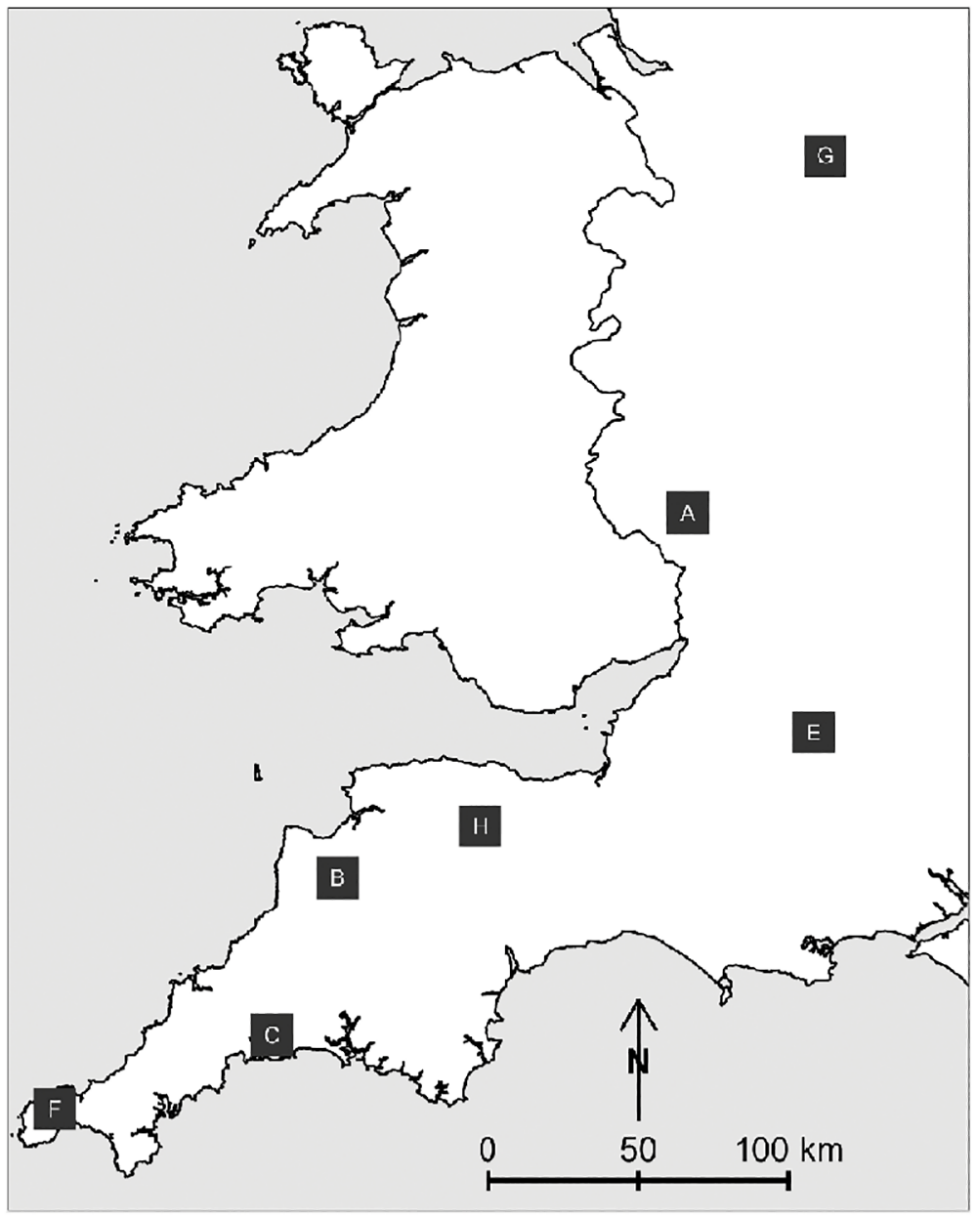
Locations of proactive trial areas of Randomised Badger Culling trial. Only the areas in which the initial cull was carried out before the trial was suspended due to the 2001 foot and mouth disease epidemic, and therefore included in this analysis, are shown.

Seven proactive trial areas from the RBCT (denoted A3, B2, C3, E3, F1, G2 and H2), in which culling commenced before the FMD epidemic, were included in this analysis. Characteristics of these areas, with results from the initial cull, are shown in Table 2, and their locations in Fig 1.

**Table 2:** Characteristics of included areas from proactive trial regions of first cull of the Randomised Badger Culling Trial.

Proactive trial region	Area of bounding box (km^2^) *	Cattle herd breakdowns †	Number of infected setts ‡	Number of uninfected setts
A3	177	15	7	21
B2	169	22	10	97
C3	99.3	12	2	72
E3	152	9	23	90
F1	13.8	4	2	16
G2	92.7	8	15	73
H2	122	16	9	53
*Total*		*86*	*68*	*422*

* Areas defined by bounding box of breakdown locations plus 5% buffer zone

^†^ Confirmed cattle herd breakdowns in one year prior to start of initial cull (excluding one outlying breakdown in area E3)

^‡^ Infected sett, a sett at which at least one TB-infected badger was captured

In the year prior to the initial cull, a total of 86 (mean 12) cattle herd breakdowns occurred across the trial regions, excluding one breakdown that was outlying. Trial areas, defined by the bounding box of the breakdown locations with a 5% buffer, ranged in size from 14 km^2^ (F1) to 177 km^2^ (A3). Within these areas, badgers were captured at a total of 490 setts. The proportion of setts that were infected ranged from 3% (C3) to 33% (A3), with 14% infected overall.

**Fig 2:**
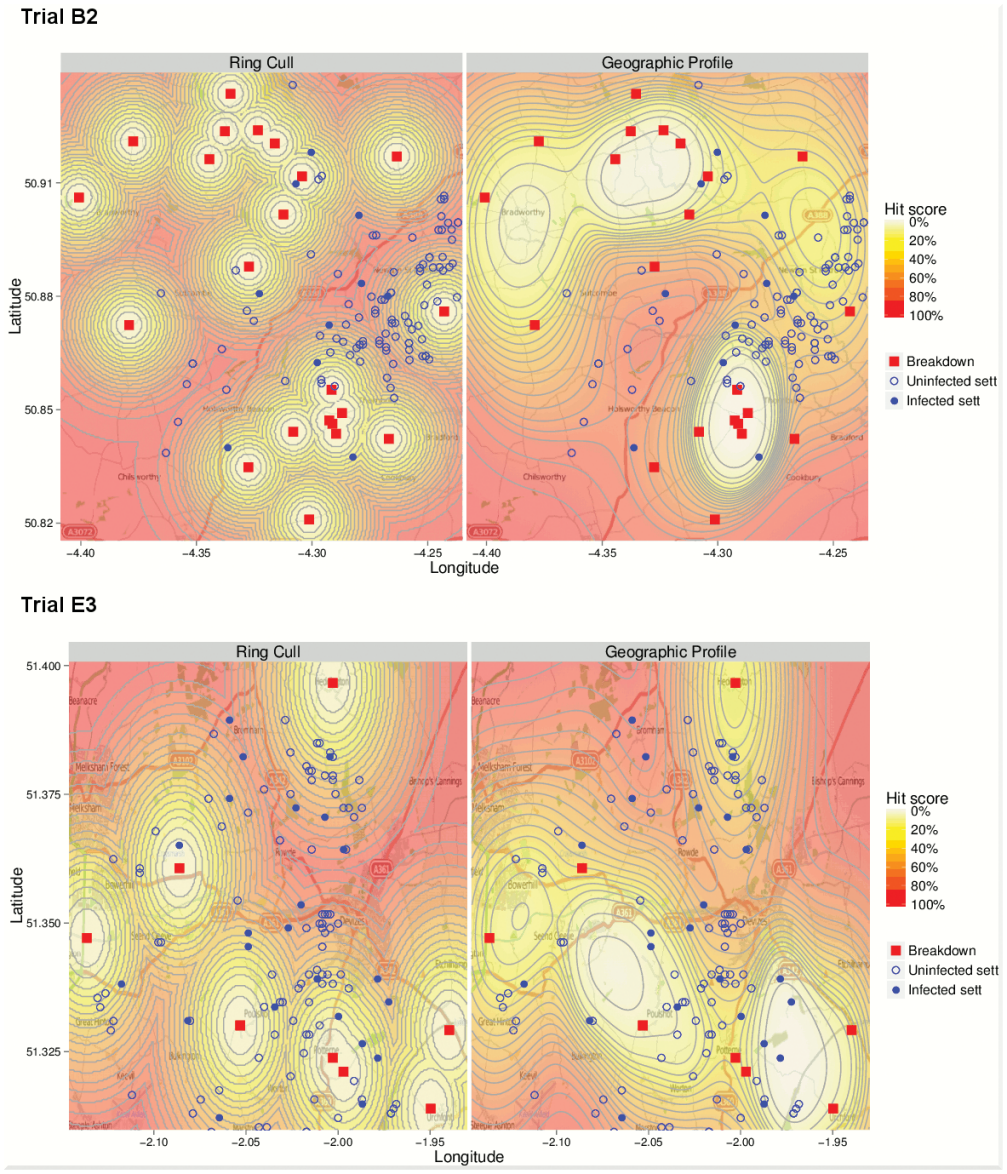
Distributions of hit scores around cattle herd breakdowns, designed using ring cull and geographic search strategies for trial regions B2 and E3.

Search strategies for setts in each trial area were designed using the GP and ring cull methods, and hit scores calculated. Example search strategies for areas B2 and E3 are shown in Fig 2. GP grouped breakdowns into clusters based on the dispersal parameter σ, producing heterogeneous distributions of hit scores with the lowest hit scores, i.e. the locations most likely to be a source, in the centre of apparent clusters of breakdowns. By contrast, the ring cull method produced symmetrically distributed hit scores around all breakdowns, and did not prioritise areas in the centre of clusters. Infected setts in trial B2 were generally located in areas with lower hit scores for the ring cull design, and in trial E3 are located in areas with lower hit scores for the GP design.

**Fig 3:**
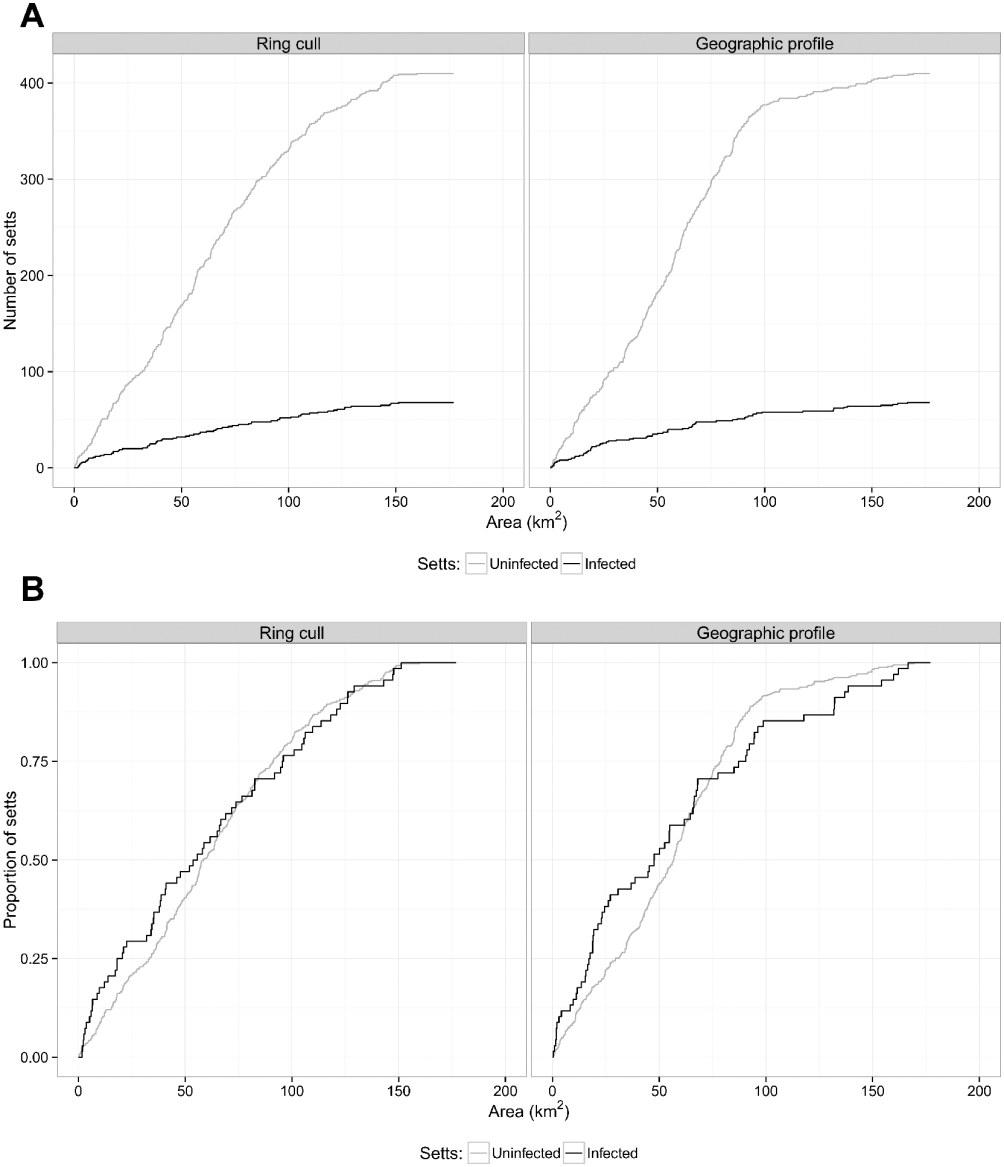
Kaplan-Meier curves comparing sizes of search areas for setts housing TB-infected and uninfected badgers, by ring cull and geographic profile methods. **A**: Numbers of TB-infected and uninfected setts; **B**: Proportions of TB-infected and uninfected setts.

Spatial survival functions were calculated to compare search strategies for infected and uninfected setts across all trial areas and search sizes. For both the ring cull and GP methods, the numbers of uninfected setts included in a cull would be much greater than for infected setts at all search sizes (Fig 3A). Table 3 presents the numbers of setts that would be included at a range of search area thresholds if each threshold was applied across all trial areas. For example, searching the highest probability 10 km^2^ of every trial area would include on average three uninfected setts by ring cull, and four by GP, for every infected sett included. The reactive culling strategy in the RBCT culled across an average of 5.3 km^2^ [9]. Searching at this threshold would include only 7 (10%) of the infected setts at the expense of 18 (4%) uninfected setts by ring cull and 8 (12%) infected and 23 (5%) uninfected setts by GP.

**Table 3:** Infected and uninfected setts included in searches with different thresholds according to search strategy, aggregated across all trial areas.

Search area threshold (km^2^)	Search strategy			
	Ring cull		Geographic profile	
	Infected*	Uninfected	Infected	Uninfected
5.3†	7	18	8	23
10	12	36	10	37
50	32	171	35	186
100	52	339	58	387
150	67	419	64	415
177 (max)	68	422	68	422

*Infected setts, setts at which at least one TB-infected badger was captured.

^†^ Average size of reactive culling operation in RBCT

Relative proportions of infected and uninfected setts included by spatially targeted culls were compared using KM curves (Fig 3B). If infected setts were identified more efficiently than uninfected setts, we would expect the KM curve for infected setts to have a steeper gradient than that for uninfected setts, locating a higher proportion of infected setts within a smaller search area. Using GP, the KM curve for the proportion of infected setts lies above the curve for uninfected setts for the first 70 km^2^ of the search, and below it for larger search areas. This implies that GP identified infected setts more efficiently than uninfected setts across smaller search areas only. Multilevel Cox regression analysis (Table 4) gave a hazard ratio (HR, infected setts/ uninfected setts) of 1.29 (95% CI 0.94–1.77, p = 0.11) for areas smaller than 70 km^2^. Therefore, for every 1 km^2^ increase in search area, the search strategy defined by the model would include a 29% higher proportion of infected than uninfected setts, but the p value indicates that this difference was not significant. For larger search areas, there was evidence that the rate of inclusion of infected setts was significantly lower than for uninfected setts (HR (infected/ uninfected setts) = 0.58, 95% CI 0.36–0.94, p = 0.03). Using the ring cull method, KM curves for infected and uninfected setts were similar (HR (infected/ uninfected setts) = 0.94, 95% CI 0.72–1.23, p = 0.64). Unadjusted Cox regression analysis produced similar results to the multilevel model for all analyses (Table 4).

**Table 4:** Cox regression spatial survival analysis comparing search strategies, adjusted for random effects of trial area.

Search strategies	Unadjusted			Multilevel*		
	HR	95% CI	p	HR	95% CI	p
**Ring cull** infected compared to uninfected setts	0.98	0.76–1.27	0.90	0.94	0.72–1.22	0.64
**Geographic profile** infected compared to uninfected setts, <70 km^2^	1.18	0.87–1.61	0.29	1.29	0.94–1.77	0.11
**Geographic profile** infected compared to uninfected setts, > = 70 km^2^	0.52	0.33–0.84	0.0074	0.58	0.36–0.94	0.03
**Infected setts** geographic profile compared to ring cull	1.00	0.71–1.40	0.99	1.03	0.73–1.45	0.87

*Multilevel Cox regression model adjusted for random effects of trial area;

HR, hazard ratio; CI, confidence interval

**Fig 4:**
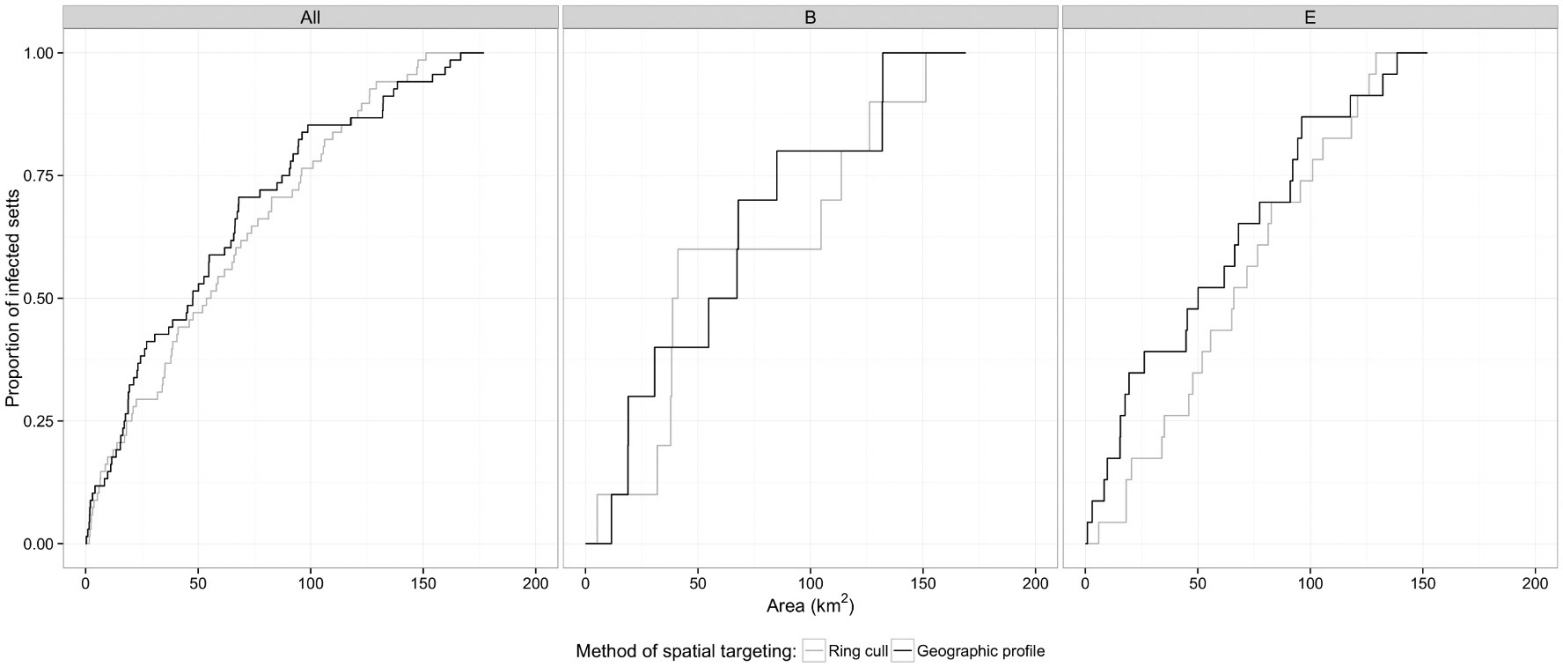
Kaplan-Meier curves comparing search areas for setts housing TB-infected badgers by ring cull and geographic profile methods for all trial regions; region B2 and region E3.

Proportions of infected setts included in search areas of increasing size using the two different methods of spatial targeting were also compared through KM plots (Fig 4). Although examination of the hit score distributions (Fig 2) suggested that the ring cull was more efficient for trial B2, and GP more efficient for trial E3, the KM curves for these areas did not differ significantly (log-rank trial B2 *p* = 0.73, trial E3 *p* = 0.66). Aggregating across all trial areas, the curves for the two approaches were not significantly different at unadjusted or multilevel Cox regression analysis (HR (GP/ ring cull) 1.03, 95% CI 0.723–1.45, p = 0.87). These curves also show that culling over very large areas would be required to capture a large proportion of infected setts. For example, including 70% infected setts would require culling over 88 km^2^ (50% total area) and 73 km^2^ (42% area) by ring cull and GP respectively. Sensitivity analyses with alternative values of the GP clustering parameter, σ, also showed no improvement of GP over ring culls.

## Discussion

In this study we used historical data from the Randomised Badger Culling Trial to assess two hypothetical methods of spatial targeting of TB-infected badgers, a simple ring cull and a novel geographic profiling approach. Targeting small areas using the GP method, which accounts for clustering, showed a small but non-significant increase in the proportion of setts with TB-infected compared to uninfected badgers included in a cull. However, GP provided no overall improvement in efficiency at targeting setts with infected badgers compared to the ring cull. Licences for current culling operations in England require that at least 70% of the badger population is culled [10]. Our results showed that both hypothetical methods would require coverage of very large areas to ensure that this proportion of infected badgers were removed, and would also result in many uninfected badgers being culled.

Spatial associations between TB infection in badgers and cattle have been demonstrated previously [1,11]. In this study we use a novel approach to assess if these spatial associations could be exploited to design an efficient spatially targeted cull so that future culls need not be conducted over such large areas. Given the controversy in the use of badger culling to control cattle TB, a culling strategy that includes a large proportion of the setts housing TB-infected badgers whilst minimising the number of setts with uninfected badgers could be favourable. Spatial targeting by a ring cull or GP would theoretically be able to achieve this by focusing the cull on areas close to cattle herd breakdowns, where a common source of infection is most likely to be located, and excluding less probable areas. However, our results showed that capture of large proportions of infected badgers could only be achieved by culling over large areas. Moreover, uninfected badgers could only be excluded from a cull by excluding substantial proportions of infected badgers. It is postulated that such culls disrupt badger social groups, causing them to range more widely, a behaviour known as perturbation, and can lead to increases in cattle TB incidence [12,29]. Therefore, any methods of localised culling that fail to include large proportions of infected badgers are not only harmful to wildlife populations but also risk the detrimental effects of perturbation.

This study used a spatial survival analysis to compare methods of spatial targeting. Survival analyses have been applied to spatial data previously [23,30], and although these examples used distance to event, rather than area to event, the same logic can be applied. Quantification of the effectiveness of the ring cull approach through spatial survival analysis clearly demonstrated the limitations of the reactive culling strategy used during the RBCT, which captured badgers within an average area of 5.3 km^2^ [9]. It is evident from the KM plots that culling over such small areas would inevitably omit large proportions of infected badgers. Such analysis could have other applications in environmental epidemiology. For example, outbreaks of infectious diseases with a suspected environmental point source have been evaluated through ‘concentric circle’ analysis in which risk of infection is compared in zones of increasing distance from each potential source [31–33]. Spatial survival analysis could enhance this method by evaluating risk across a continuous range of search sizes, rather than requiring selection of arbitrary thresholds.

Ring culling approaches have been used for the control of other diseases of agricultural importance, notably foot and mouth disease, a viral infection of many cloven-footed mammals including cattle and sheep. In 2001, large scale ring culling was implemented in response to epidemics of FMD in the UK and the Netherlands with the aim of eliminating animals incubating infections that may have spread from the outbreak farms [34]. Modelling during the early phase of the epidemic informed these measures which, in the worst affected regions of the UK, involved culling all sheep within 3 km of an infected farm [35]. Through combination with other control measures, the epidemics were eventually brought under control. However, the ring cull was very expensive and issues included logistical implications of removal of dead animals, risk of spread of infection during the culling operations, and selection of the appropriate size and shape of the cull [34]. This highlights the difficulty of implementing a ring cull policy, even in a situation such as the FMD epidemic, in which the clear focal nature of the disease and the absence of an intermediate host strengthened the rationale for the approach.

GP methods have been shown to identify sources of infectious diseases efficiently in other settings. A series of cases of malaria in Cairo were used to identify six out of seven mosquito breeding sites within 2% of the search area, outperforming other measures of spatial central tendency; and the technique also performed well when applied to cases of cholera in John Snow’s classic study [36].

An important factor that limited the success of spatial targeting in this study is the uncertainty in the degree to which cattle TB results from badger to cattle transmission. An estimate using data from the RBCT suggested that contribution of badgers to disease in cattle is approximately 50%, but that only around 5–7% of this was a result of direct badger to cattle transmission, with the remainder due to amplification of these events through onward cattle to cattle transmission [37]. Higher contributions of localised sources have also been suggested from other models [38]. Our results support low estimates, as we would expect GP to perform more effectively than a ring cull if the majority of TB was transmitted from badgers to cattle resulting in distinct clustering. Molecular data could be used to test this hypothesis by comparing the strain types of TB in cattle to those in badgers in high and low probability areas of the geographic profile, although it could not confirm the direction of transmission.

Another limitation to this data was that spatial analyses were conducted using point locations for cattle herds. These locations are clearly a simplification of the true area that the cattle herds occupied which may have spanned several fields during the study period, and could have altered the apparent clustering of the breakdowns. Furthermore, a number of farms were composed of more than one non-conjoint land parcel, meaning that cattle herds can be located several kilometres from the farm. More detailed information on the locations of herds at different times could potentially refine the analysis, but, given that the time of infection is difficult to determine, a high level of accuracy in exposure location is unlikely to be achieved.

Misclassification of badger and cattle TB status could also have reduced the apparent success of spatial targeting in this study. Proactive culling trial regions of the RBCT were used in this analysis because badgers were culled, and therefore tested for TB, across the entire area. However, it is possible that some areas were identified by spatial targeting as likely source locations in which infected badgers were located but were not identified during the RBCT. This could be because badgers had migrated since infecting cattle, evaded capture at setts, or were captured but misclassified as uninfected at post mortem due to deficiencies in diagnostic procedures. The sensitivity of the standard post-mortem to detect TB in badgers is estimated to be not more than 55% [18]. Similarly, the sensitivity of the tuberculin skin test at the herd level may be as low as 50% if only one animal is infected [5,39], so some herds may have been misclassified as uninfected, leading to an incomplete representation of the distribution of TB in cattle herds. Furthermore, some TB breakdowns could have been caused by cattle moving into a herd and not through contact with badgers. Accurate identification breakdowns of badger origin, through information on cattle movement and molecular typing, could improve the power of spatial targeting methods [40].

A challenge of applying GP analysis to a new biological system is the selection of an appropriate value for the dispersal parameter, σ. Here, we used a value of 0.02 decimal degrees, which assumed that 68% of breakdowns arising from badger-cattle transmission would have occurred within approximately 1700m of infected setts. This value was chosen to allow potential dispersal of badgers to the upper limit of their likely home range sizes, which have generally had smaller estimates in the areas where the RBCT was conducted [9,20,21]. We conducted sensitivity analyses using a range of alternative dispersal parameter values, and also found no improvement over ring culling.

Another limitation of the GP approach is that it requires a rectangular search area to be defined, which inevitably includes some areas that could never harbour a source. In this example, search areas would ideally match exactly the areas surveyed for badger setts during the RBCT, and therefore exclude, for example, stretches of water and urban areas. We used the same search areas to analyse the ring cull so that valid comparisons between methods could be made, but it would be useful to refine the GP model to allow exclusion of unsuitable land through integration with geographic information systems.

In summary, we found that cattle TB incidents were insufficiently clustered around TB-infected badger setts to define an efficient spatially targeted badger culling strategy. The hypothetical targeting methods tested did not effectively prioritise areas populated with TB-infected badgers over areas with uninfected badgers. Further research in other areas, such as improvements in detection of infection and direction of transmission, is therefore necessary if the numbers of healthy badgers culled for bovine TB control is to be limited.

## References

[1] Krebs JR, Anderson R, Clutton-Brock T, Morrison I, Young D, Donnelly CA (1997) Bovine tuberculosis in cattle and badgers. London: Ministry of Agriculture, Fisheries and Food (MAFF) Publications.

[2] Department for Environment Food and Rural Affairs (2015) Monthly publication of National Statistics on the Incidence of Tuberculosis (TB) in Cattle to end December 2014 for Great Britain.

[3] Department for Environment Food and Rural Affairs (2012) Changes to TB Cattle Movement Controls. London.

[4] Murhead RH, Burns KJ (1974) Tuberculosis in wild badgers in Gloucestershire: epidemiology. Veterinary Record 95. 10.1136/vr.95.24.552 4462735

[5] Godfray HC, Donnelly CA, Kao RR, Macdonald DW, McDonald RA, Petrokofsky G, et al (2013) A restatement of the natural science evidence base relevant to the control of bovine tuberculosis in Great Britain. Proc Biol Sci 280: 20131634 10.1098/rspb.2013.1634 23926157 PMC3757986

[6] Dunnet GM, Jones DM, McInerney JP (1986) Badgers and Bovine Tuberculosis: Review of Policy. London: HMSO.

[7] Zuckerman S (1980) Badgers, Cattle and Tuberculosis. London: HMSO.

[8] Department for Environment Food and Rural Affairs (2014) The Strategy of achieving Officially Bovine Tuberculosis Free status for England.

[9] Bourne FJ, Donnelly CA, Cox DR, Gettinby G, McInerney JP, Morrison WI, et al. (2007) Final Report of the Independent Scientific Group on Cattle TB. 10737292

[10] Independent Expert Panel (2014) Pilot Badger Culls in Somerset and Gloucestershire. London.

[11] Woodroffe R, Donnelly CA, Johnston WT, Bourne FJ, Cheeseman CL, Clifton-Hadley RS, et al (2005) Spatial association of Mycobacterium bovis infection in cattle and badgers Meles meles. Journal of Applied Ecology 42: 852–862.

[12] Woodroffe R, Donnelly CA, Cox DR, Bourne FJ, Cheeseman CL, Delahay RJ, et al (2006) Effects of culling on badger Meles meles spatial organization: implications for the control of bovine tuberculosis. Journal of Applied Ecology 43: 1–10.

[13] Donnelly CA, Woodroffe R, Cox DR, Bourne J, Gettinby G, Le Fevre AM, et al (2003) Impact of localized badger culling on tuberculosis incidence in British cattle. Nature 426: 834–837. 14634671 10.1038/nature02192

[14] Vial F, Donnelly CA (2012) Localized reactive badger culling increases risk of bovine tuberculosis in nearby cattle herds. Biol Lett 8: 50–53. 10.1098/rsbl.2011.0554 21752812 PMC3259956

[15] Rossmo DK (2000) Geographic Profiling. Boca Raton, Florida, USA: CRC Press.

[16] Le Comber SC, Stevenson MD (2012) From Jack the Ripper to epidemiology and ecology. Trends Ecol Evol 27: 307–308. 10.1016/j.tree.2012.03.004 22494610

[17] Woodroffe R, Donnelly CA, Jenkins HE, Johnston WT, Cox DR, Bourne FJ, et al (2006) Culling and cattle controls influence tuberculosis risk for badgers. Proc Natl Acad Sci U S A 103: 14713–14717. 17015843 10.1073/pnas.0606251103 PMC1586183

[18] Crawshaw TR, Griffiths IB, Clifton-Hadley RS (2008) Comparison of a standard and a detailed postmortem protocol for detecting Mycobacterium bovis in badgers. Veterinary Record 163: 473–477. 18931354 10.1136/vr.163.16.473

[19] Verity R, Stevenson MD, Rossmo DK, Nichols RA, Le Comber SC (2014) Spatial targeting of infectious disease control: identifying multiple, unknown sources. Methods in Ecology and Evolution: 5: 647–655.

[20] Rogers LM, Cheeseman CL, Mallinson PJ, Clifton-Hadley R (1997) The demography of a high-density badger (Meles meles) population in the west of England. Journal of Zoology 242: 705–728.

[21] Kauhala K, Holmala K (2011) Landscape features, home-range size and density of northern badgers (Meles meles). Annales Zoologici Fennici 48: 221–232.

[22] Pope LC, Butlin RK, Wilson GJ, Woodroffe R, Erven K, Conyers CM, et al (2007) Genetic evidence that culling increases badger movement: implications for the spread of bovine tuberculosis. Molecular Ecology 16: 4919–4929. 17944854 10.1111/j.1365-294X.2007.03553.x

[23] Carruthers JI, Lewis S, Knaap G-J, Renner RN (2010) Coming undone: A spatial hazard analysis of urban form in American metropolitan areas*. Papers in Regional Science 89: 65–88.

[24] R Core Team (2013) R: A language and environment for statistical computing. R Foundation for Statistical Computing, Vienna, Austria.

[25] Bivand R, Rundel C (2014) rgeos: Interface to Geometry Engine—Open Source (GEOS). 0.3–3 ed.

[26] Stevenson MD, Verity R (2013) Rgeoprofile: Generates geograpic profiles from point pattern data. 1.1 ed.

[27] Therneau T (2014) A Package for Survival Analysis in S. A Package for Survival Analysis in S ed.

[28] Therneau T (2012) coxme: Mixed Effects Cox Models.

[29] Bielby J, Donnelly CA, Pope LC, Burke T, Woodroffe R (2014) Badger responses to small-scale culling may compromise targeted control of bovine tuberculosis. Proceedings of the National Academy of Sciences 111: 9193–9198. 10.1073/pnas.1401503111 PMC4078854 24927589

[30] Reader S (2000) Using survival analysis to study spatial point patterns in geographical epidemiology. Soc Sci Med 50: 985–1000. 10714921 10.1016/s0277-9536(99)00349-4

[31] Brown CM, Nuorti PJ, Breiman RF, Hathcock AL, Fields BS, Lipman HB, et al (1999) A community outbreak of Legionnaires' disease linked to hospital cooling towers: an epidemiological method to calculate dose of exposure. International Journal of Epidemiology 28: 353–359. 10342703 10.1093/ije/28.2.353

[32] Nguyen TM, Ilef D, Jarraud S, Rouil L, Campese C, Che D, et al (2006) A community-wide outbreak of legionnaires disease linked to industrial cooling towers—how far can contaminated aerosols spread? Journal of Infectious Diseases 193: 102–111. 16323138 10.1086/498575

[33] Schimmer B, Veenstra T, Ter Schegget R, Wegdam-Blans M, Zuchner L, De Bruin A, et al (2010) The use of a geographic information system to identify a goat dairy farm as the most likely source of an Urban Q fever outbreak. Clinical Microbiology and Infection 16: S391–S392. 10.1186/1471-2334-10-69 PMC2848044 20230650

[34] Sutmoller P, Barteling SS, Olascoaga RC, Sumption KJ (2003) Control and eradication of foot-and-mouth disease. Virus Res 91: 101–144. 12527440 10.1016/s0168-1702(02)00262-9

[35] Ferguson NM, Donnelly CA, Anderson RM (2001) The foot-and-mouth epidemic in Great Britain: pattern of spread and impact of interventions. Science 292: 1155–1160. 11303090 10.1126/science.1061020

[36] Le Comber SC, Rossmo DK, Hassan AN, Fuller DO, Beier JC (2011) Geographic profiling as a novel spatial tool for targeting infectious disease control. Int J Health Geogr 10: 35 10.1186/1476-072X-10-35 21592339 PMC3123167

[37] Donnelly CA, Nouvellet P (2013) The contribution of badgers to confirmed tuberculosis in cattle in high-incidence areas in England. PLoS Currents. 10.1371/currents.outbreaks.097a904d3f3619db2fe78d24bc776098 PMC3992815 24761309

[38] Green DM, Kiss IZ, Mitchell AP, Kao RR (2008) Estimates for local and movement-based transmission of bovine tuberculosis in British cattle. Proc Biol Sci 275: 1001–1005. 10.1098/rspb.2007.1601 18252669 PMC2366193

[39] Conlan AJ, McKinley TJ, Karolemeas K, Pollock EB, Goodchild AV, Mitchell AP, et al (2012) Estimating the hidden burden of bovine tuberculosis in Great Britain. PLoS Comput Biol 8: e1002730 10.1371/journal.pcbi.1002730 23093923 PMC3475695

[40] Ramos DF, Tavares L, da Silva PE, Dellagostin OA (2014) Molecular typing of Mycobacterium bovis isolates: a review. Braz J Microbiol 45: 365–372. 25242917 10.1590/s1517-83822014005000045 PMC4166258

